# Effect of Thymol Addition and Withdrawal on Some Blood Parameters, Antioxidative Defence System and Fatty Acid Profile in Rabbit Muscle

**DOI:** 10.3390/ani10081248

**Published:** 2020-07-22

**Authors:** Kristina Bacova, Karin Zitterl-Eglseer, Lubica Chrastinova, Andrea Laukova, Michaela Madarova, Sona Gancarcikova, Drahomira Sopkova, Zuzana Andrejcakova, Iveta Placha

**Affiliations:** 1 Centre of Biosciences, Slovak Academy of Sciences, Institute of Animal Physiology, Soltesovej 4–6, 040 01 Kosice, Slovakia; bacovak@saske.sk (K.B.); laukova@saske.sk (A.L.); madarova@saske.sk (M.M.); 2 Institute of Animal Nutrition and Functional Plant Compounds, University of Veterinary Medicine Vienna, Veterinärplatz 1, A-1210 Wien, Austria; karin.zitterl@vetmeduni.ac.at; 3 National Agricultural and Food Centre, Hlohovecka 2, 951 41 Nitra-Luzianky, Slovakia; lubica.chrastinova@nppc.sk; 4 Laboratory of Gnotobiology, Department of Microbiology and Immunology, University of Veterinary Medicine and Pharmacy, Komenskeho 73, 041 81 Kosice, Slovakia; sona.gancarcikova@uvlf.sk; 5 Department of Anatomy, Histology and Physiology, University of Veterinary Medicine and Pharmacy, Komenskeho 73, 041 81 Kosice, Slovakia; drahomira.sopkova@uvlf.sk (D.S.); zuzana.andrejcakova@uvlf.sk (Z.A.)

**Keywords:** rabbit, thymol, bioavailability, antioxidant

## Abstract

**Simple Summary:**

So far, the study of the bioactivity of thymol, a major constituent of *Thymus vulgaris* L., in the animal organism has received little attention. Our study could give us answers to questions about whether thymol accumulates in the rabbit organism after its sustained administration and if it is also able to exhibit its beneficial properties for a longer period. Thymol in powder form at the concentration 250-mg/kg feed was added to the rabbit diet for 21 days and withdrawn for the next seven days. We confirmed that thymol was sufficiently absorbed from the gastrointestinal tract and was able to express its biological activity not only during application but, also, after withdrawal. Further studies are needed to clarify the biotransformation and bioavailability of thymol in the rabbit organism with respect to the specific features of rabbit digestion.

**Abstract:**

Thymol concentrations in rabbit plasma, intestinal wall (IW) and faeces were detected, and the effects of thymol application and withdrawal on biochemical, antioxidant parameters and fatty acids (FA) in blood (B) and muscle (M) were studied. Forty-eight rabbits were divided into two experimental groups (control, C and with thymol 250-mg/kg feed, T). Thymol was administered for 21 days (TA) and withdrawn for seven days (TW). Thymol in plasma correlated with that in the IW (Spearman′s correlation coefficient (r_s_) = −1.000, *p* = 0.0167, TA) and was detected in faeces (TA and TW). In TA alkaline phosphatase (*p* = 0.0183), cholesterol (*p* = 0.0228), malondialdehyde (*p* = 0.003), glutathione peroxidase (*p* = 0.0177) in B and lactate dehydrogenase (M, *p* = 0.0411) decreased; monounsaturated FA (*p* = 0.0104) and α-linolenic acid (*p* = 0.0227) in M increased. In TW urea (*p* = 0.0079), docosapentaenoic acid (*p* = 0.0069) in M increased; linoleic acid (*p* = 0.0070), ∑ n−6 (*p* = 0.0007) in M and triglycerides decreased (B, *p* = 0.0317). In TA and TW, the total protein (*p* = 0.0025 and 0.0079), creatinine (B; *p* = 0.0357 and 0.0159) and oleic acid (M; *p* = 0.0104 and 0.0006) increased. Thymol was efficiently absorbed from the intestine and demonstrated its biological activity in blood and the muscles.

## 1. Introduction

The supplementation of human and animal diets with *Thymus vulgaris* (thyme) either as dried leaves or its essential oil has often demonstrated its beneficial properties. Since synthetic growth promoters were replaced with alternative herbal products in animal rearing, thyme has started receiving major attention. The most important bioactive compound contained in this plant is thymol, which exhibits antimicrobial, antioxidant, anticarcinogenic and anti-inflammatory activities [1,2,3]. According to some studies, thyme improves the performance parameters, but some other studies suggest that it has no effect. Some studies report that thyme is able to reduce levels of triglyceride and total cholesterol [4,5]. Yu et al. [6] found that thymol possesses a lipid-reducing function by altering hepatic triglyceride secretion.

The mode of action of herbs and plant extracts and the details about the accumulation of phenolic substances in animal tissues are not known or not completely understood [7,8]. In general, the bioavailability of dietary compounds depends on their digestive stability and the efficiency of their transepithelial passage [9]. Moreover, their influence on each other′s intestinal absorption has to be taken into account in studies concerned with the bioavailability of essential oil compounds.

Thymol as one of the major constituents of thyme oil presents a wide range of functional possibilities in pharmacy and the food industry. Besides thymol, thyme contains high concentrations of monoterpene phenols like carvacrol, p-cymene, 1,8-cineole, linalool, borneol, camphor, β-caryophyllene, thymol methyl ether and carvacrol methyl ether, which could have influenced the thymol absorption [2,3].

Oceľová et al. [10] were the first who analysed the thymol concentrations in individual intestinal segments in hens. Sufficient absorption of thymol from the digestive tract and its transport by systemic circulation to tissues in broiler chickens after four weeks of thyme essential oil application were demonstrated by Oceľová et al. [7]. Placha et al. [2] pointed to the sparing effect of thymol against oxidative deterioration of the antioxidant defence system in poultry after sustained thyme oil dietary application at 0.05% (thymol content 248.97 mg/kg dry matter (DM)) and 0.1% (thymol content 460.22 mg/kg DM) concentrations.

Concerning the toxicity of thymol, the data are controversial. According to a recent report of the European Food Safety Authority (EFSA) [11], thymol, when administered by the oral route in a rat, mouse and guinea pig (lethal dose - LD 50, 0.98, 1.80 and 0.88 mg/kg, respectively), suggested a moderately acute toxicity. No significant alteration was observed when a thymol oil-water emulsion was administered at doses (15.39, 30.78 and 61.55 mg/kg, respectively) during 28 days [12]. Based on European Commission (EC) [13], no maximum residual limit for thymol in foodstuffs of animal origin is needed to establish when it is used as a veterinary medicinal product. These data show that further studies are required to establish the thymol appropriate concentration.

Rabbits have a unique digestive system that is represented by an original feature of rabbit feeding behaviour named caecotrophy. Caecotrophy means the excretion and immediate consumption of specific soft faeces termed “caecotrophs”. This process is extremely important, because it improves feed utilization by maximizing the digestibility of nutrients [14]. Many scientific studies have tried to find the appropriate dose and form of plant extract application for improving animal health, but insights into the precise mechanism or mode of action of their components are lacking. As far as rabbits are concerned, to our knowledge, only one old study by Takada et al. [15] has been carried out regarding the metabolic outcome of thymol. Probably, the process of caecotrophy could minimize the loss of thymol bioaccessibility during the digestive processes.

The present study provides new insights for understanding the possible processes of absorption and deposition of thymol in the rabbit organism in connection with its protective role against oxidative stress. Based on our previous studies related to the thymol absorption, deposition and beneficial effects against oxidative stress in broiler chickens after a sustained administration of thyme oil, we decided to examine the thymol application into rabbit diets at the concentration 250-mg/kg feed.

## 2. Materials and Methods

### 2.1. Animals and Experimental Design

After weaning at 35 days of age, 48 rabbits of both sexes (meat line M9) were randomly divided into two experimental groups (control, C and with thymol addition, T), with six replicates in each (one replicate consisting of two cages, one cage/two animals). Initial live weight was 1006 ± 98 g in C and 1035 ± 107 g in T. All experimental wire-net cages (61 cm × 34 cm × 33 cm) were kept in rooms with automatic temperature control (22 ± 4 °C) and photoperiod (16 L:8 D). The rabbits could feed ad libitum and had free access to drinking water. The experiment lasted 28 days. The rabbits were fed with thymol addition for 21 days (TA), and for the next 7 days, the thymol was withdrawn (TW). Eight starved rabbits (6 male and 2 female) in each group were slaughtered in an experimental slaughterhouse at 56 (C and TA) or 63 (C and TW) days of age. Rabbits were stunned with electronarcosis (50 Hz, 0.3 A/rabbit for 5 s), immediately hung by the hind legs on the processing line and quickly bled by cutting the jugular veins and the carotid arteries.

### 2.2. Animals Care and Use

The trial was carried out at the experimental rabbit facility of the National Agricultural and Food Centre, Research Institute for Animal Production, Nitra, Slovakia. The protocol was approved by the Institutional Ethical Committee, and the State Veterinary and Food Office of the Slovak Republic approved the experimental protocol (4047/16-221).

### 2.3. Diets and Chemical Analyses

The basal diet (control, C) was formulated to satisfy growing rabbits′ requirements [16] (Table 1) and was tested against the experimental diet (T) containing thymol (≥99.9%, Sigma Aldrich, St. Louis, MO, USA), which was added to the basal diet in white powder form at concentration 250-mg/kg feed. The diets were administered in the form of pellets with an average size of 3.5 mm. The feed was stored in darkness to protect against degradative processes and was analysed to determine the crude protein (CP), ash, ether extract, acid detergent fibre (ADF), starch and dry matter (DM) in the diets, while DM was also determined for the intestinal wall, muscle, liver and faeces according to the Association of Official Analytical Chemists (AOAC) methods [17]. Neutral detergent fibre (NDF) was analysed according to Van Soest et al. [18].

### 2.4. Growth Performance and Health Status

Body weight (BW) and feed intake (FI) were recorded individually once a week. The average daily FI, average daily weight gain (WG) and feed conversion ratio (FCR) were calculated at the end of the trial (on 56 and 63 d of age). Data pertaining to any animal that died during the experiment were excluded from the calculations. Mortality was recorded daily throughout the experimental periods.

### 2.5. Thymol Antioxidant Capacity and Stability in Feed

The Trolox equivalent capacity (TEAC) was determined in thymol and in the experimental feed according with the method described by Karamać et al. [19] using the 2,2’ — Azinobis — (3 — Ethylbenzthiazolin — 6 — Sulfonic Acid (ABTS •+) decolorization assay. The results were expressed as mmol Trolox equivalents (TE) per g. Thymol evaporation in the feed was analysed every week during thymol application using High — Performance Liquid Chromatography HPLC according to the modified method of Pisarčíková et al. [20]. Samples were analysed in triplicate and were relatively stable (0 d—151.89, 7 d—134.75 and 14 d—128.30 µg/g DM, respectively).

### 2.6. Sampling

Blood samples for biochemical analyses were collected from the marginal ear vein (Vena auricularis) into dry nonheparinized Eppendorf tubes at experimental days 21 and 28 and were left to clot in a standing position for approximately 2 h to obtain the serum, and then, the serum was separated by centrifugation at 700× *g* for 15 min. Blood for analyses of antioxidant parameters was collected into heparinized tubes, and plasma was obtained after centrifugation at 1180× *g* for 15 min. Samples of serum, plasma, muscle (*Longissimus thoracis et lumborum*, LTL), small intestinal wall, liver and hard faeces (freshly voided, collected using nets mounted under the cages) were immediately frozen in liquid nitrogen and stored at −70 °C until analysis.

### 2.7. Thymol Analyses in Plasma, Small Intestinal Wall and Faeces

Detection of thymol in samples of plasma, intestinal walls and faeces was performed using headspace solid-phase microextraction followed by gas chromatography coupled with the mass spectrometry method, as described by Placha et al. [2]. Briefly, detection and quantification were carried out using a gas chromatography/mass spectrometry (GC/MS) (type HP 6890 GC) coupled with a 5972 quadrupole-mass selective detector (Agilent Technologies GmbH, Wilmington, DE, USA). Detection of thymol was confirmed by comparing its specific mass spectrum and retention time with those of the reference compound. Additionally, the Kovats index was calculated. Calibration curves were generated by plotting the peak area ratios of thymol to o-cresol used as the internal standard (Sigma-Aldrich, St. Louis, MO, USA) against the known thymol concentrations. The selective ion mode was used for the quantitative analysis of thymol. The mass fragments *m*/*z* 135 and *m*/*z* 150, as well as *m*/*z* 107 and *m*/*z* 108, were monitored as characteristic for thymol and o-cresol, respectively. Calibration curves were prepared from blank samples spiked directly with thymol (AppliChem, Darmstadt, Germany) in standard solutions with known concentrations. Each point on the calibration curve was analysed as a duplicate. The peak of thymol was detected around 19 min, and the o-cresol peak occurred around 10 min in all samples. Samples for the detection of thymol were prepared using the method described by Oceľová et al. [10]. Enzyme β–Glucuronidase Helix pomatia Type HP-2 (aqueous solution, ≥100,000 units/mL, Sigma-Aldrich, St Louis, MO, USA) was added to samples to cleave thymol from its glucuronide and sulphate to obtain the total amount of thymol in the plasma.

### 2.8. Biochemical and Antioxidant Parameters and Activity of Lactate Dehydrogenase in Blood and Tissues

Total proteins (TP; g/L), creatinine (µmol/L), urea (mmol/L), triglycerides (mmol/L), total cholesterol (mmol/L), alanine aminotransferase (ALT; µkat/L), aspartate aminotransferase (AST; µkat/L) and alkaline phosphatase (ALP; µkat/L) were analysed using a DIALAB commercial kit (Prague, Czech Republic) and an ELLIPSE analyser (AMS, Guidonia, Rome, Italy).

Activity of glutathione peroxidase (GPx, EC 1.11.1.9) in blood was measured by monitoring the oxidation of Nicotinamide Adenine Dinucleotide Phosphate (NADPH) at 340 nm in-line with Paglia and Valentine [21] using a commercial kit (Ransel, Randox, London, UK). Haemoglobin (Hb) content in blood was analysed using a commercial kit from Randox, UK. The samples of LTL muscle and liver for malondialdehyde (MDA) measurement and activity of lactate dehydrogenase (LDH, EC 1.1.1.27) were washed in buffered saline to remove excess blood and connective tissue. Samples for MDA analyses were homogenised with deionized distilled water and 50 µL of 7.2% butylated hydroxytoluene and for LDH activity in cold buffer (0.05-mol/L Tris-HCl buffer, pH 7.3). The homogenates were subsequently centrifuged at 13,680× *g* at 4 °C for 20 min. MDA concentrations in these tissues and plasma were measured using the modified fluorometric method of Jo and Ahn [22]. The enzyme activity of LDH was measured using a commercial diagnostic kit (Crumlin, Randox, UK) with an Alizé automatic biochemical analyser (Lisabio, Pouilly-en-Auxois, France) at 340 nm, as described by Andrejčáková et al. [23]. The protein concentration in the muscle and liver was quantified using the spectrophotometric method published by Bradford [24].

### 2.9. Fatty Acids in Muscle Tissue

The fatty acid (FA) composition in the muscle tissue was determined using the method of Ouhayoun et al. [25]. Fatty acid methyl esters (FAME) were prepared by means of alcoholises in an essential nonalcoholic solution and analysed using gas chromatography on GC 6890N (Agilent Technologies, Basel, Switzerland). Results were expressed as percentages of total fatty acids.

### 2.10. Statistical Analyses

Values of thymol, GPx, LDH, MDA and FA concentrations were tested for normal distribution with the Kolmogorov-Smirnov test. The Mann-Whitney U test was used for statistical analysis. Results were presented as the mean value ± standard deviation. Significant differences were considered at *p* < 0.05. Correlations of thymol concentrations between plasma and feed and plasma and intestinal wall were analysed using nonparametric Spearman′s rank correlation and expressed as Spearman′s correlation coefficient (r_s_). Statistical analyses were performed using Graph Pad Prism 5.0. (GraphPad Software, San Diego, CA, USA).

## 3. Results

### 3.1. Growth Performance

All broiler rabbits in the present trial were in good health, and the growth performance was normal and was not affected by the addition of thymol. Five animals (three/control and two/experimental group) died during the whole experiment. If there were no differences in the given parameters, we do not include them in the table.

### 3.2. Thymol Antioxidative Capacity

Approximately equal TEAC value was found in thymol and in the feed of the experimental group (0.76 vs. 0.74 mmol TE/g).

### 3.3. Thymol in Feed, Plasma, Small Intestinal Wall and Faeces

Thymol content in feed amounted to 148.9 ± 16.7 µg/g DM. Thymol concentration in plasma was 0.05 ± 0.02 µg/L and, in intestinal wall, 0.04 ± 0.03 µg/g DM in TA, but, in TW, it was not detected. Thymol concentration in faeces in TA was 0.89 ± 0.45 µg/g DM, and, in TW, it was 0.08 ± 0.04 µg/g DM (Table 2). Thymol concentration in plasma significantly correlated with the thymol concentration in the intestinal wall (r_s_ = −1.000, *p* = 0.0167).

### 3.4. Biochemical Parameters in Blood

Thymol addition significantly decreased the levels of ALP (*p* = 0.0183) and cholesterol (*p* = 0.0228). Urea significantly increased (*p* = 0.0079), and triglycerides decreased (*p* = 0.0317) in TW. TA and TW had significantly increased TP (*p* = 0.0025 vs. *p* = 0.0079) and creatinine (*p* = 0.0357 vs. *p* = 0.0159) (Table 3).

### 3.5. Antioxidant Parameters in Blood and Tissues

MDA and GPx in blood (*p* = 0.003 and *p* = 0.0177) and LDH in muscle (*p* = 0.0411) significantly decreased in TA (Table 4).

### 3.6. Fatty Acids in Muscle

Concentrations of oleic acid (C 18:1 n-9) significantly increased in TA and TW (*p* = 0.0104 vs. 0.0006). MUFA and α-linolenic acid (C 18:3 n-3) significantly increased in TA (*p* = 0.0104 and *p* = 0.0227). Docosapentaenoic acid (C 22:5 n-3) significantly increased (*p* = 0.0069), and linoleic acid (C 18:2 n-6) and ∑ n-6 significantly decreased in TW (*p* = 0.0070 and *p* = 0.0007; Table 5).

## 4. Discussion

### 4.1. Growth Performance

The inclusion of thymol at the concentration used in our experiment did not show any significant effect on the animals′ weight, weight gain and conversion ratio. Although thyme has been shown to improve the palatability and feed intake in growing rabbits, its beneficial effects on the live growth performance in rabbits has not yet been confirmed [26]. According to Erdelyi et al. [27], due to the specific digestive physiology of rabbits, essential oils or plant extracts have much lesser positive effects than in broilers or piglets. Windisch et al. [28] reported that the use of thyme in animal diets could be limited when applied in certain amounts, because it is highly aromatic. According to Gerencsér et al. [29], thyme leaves did not demonstrate any substantial effects on the growth performance or health status. This statement is in agreement with our study, in that the thymol did not affect the feed intake, and most animals remained in optimal condition.

### 4.2. Thymol in Plasma, Intestinal Wall and Faeces

We found a significant correlation between thymol levels in the small intestinal wall and plasma during the period of thymol addition to the feed. This result is in agreement with Placha et al. [2], who confirmed the efficient absorption of thymol from the digestive tract into the systemic circulation in broiler chickens. According to Oceľová [30], after the absorption of plant compounds from the intestine, they are metabolised and eliminated from the organism.

One of the most original features of rabbit-feeding behaviour is caecotrophy. The result of this process, in which soft faeces are swallowed and then stored intact in the fundus of the stomach, is that they undergo the same digestive processes as normal feed. Some parts of the initial food intake may be recycled even up to four times in this way [31]. The detection of thymol in faeces points to its excretion from the organism in an unmetabolised form (Table 2). This unmetabolised thymol could be recycled and again absorbed in the intestinal wall, where it is finally metabolised by the processes of biotransformation. The part of the metabolites in the intestinal wall can be transported back into the intestinal lumen by efflux transporters, converted to thymol (parental compound) and again reabsorbed by the enterocytes. Another molecule can be transported through the basolateral membrane of enterocytes directly to the blood circulation. All these processes play a crucial role in affecting the thymol concentration in blood [7].

Thymol sulphate and thymol glucuronide are the main metabolites of thymol biotransformation, and so far, little is known about their bioactivity or whether these compounds are only inactive forms [3]. Pisarčíková et al. [20] first detected thymol sulphate and thymol glucuronide in the liver and duodenal wall of broiler chickens after a sustained four-week administration of thyme essential oil, and according to Kohlert et al. [32] and Rubió et al. [9], thymol metabolites could be deconjugated to the parental compounds and, in this way, express their pharmacological properties. We have to bear in mind that, during biotransformation, plant compounds change their pharmacological properties, which usually differ from the properties of the parental compounds.

There are only a few studies concerning thymol distributions in animal tissues. Thymol conjugates have been detected in the plasma of humans and animals, and thymol after enzymatic cleavage was detected in the plasma of horses, chickens and pigs [1,10,32,33,34,35]. According to Placha et al. [2], erythrocytes can act as depots of polyphenols due to their ability to bind to the surface of red blood cells, and in this way, they can circulate in the organism. There is the question whether bound thymol is able to unbind from tissue depots if the amount of circulated thymol decreases. Since we detected thymol in rabbit faeces also after its withdrawal, this mechanism could be indicated (Table 2).

### 4.3. Biochemical Parameters in Blood

Despite the fact that plant bioactive compounds undergo fast biotransformation and elimination, Oceľová et al. [10] and Placha et al. [2] confirmed the accumulation of thymol in the breast muscles, kidneys and livers of broiler chickens after four weeks of thyme essential oil diet supplementation. Moreover, they found the highest concentration of thymol in the kidneys and the lowest in liver tissues, which could point to intensive metabolism in the liver and accumulation in the kidneys. Bardal et al. [36] showed that the distribution of plant compounds from systemic circulation to the tissues is restricted, since only free drugs in plasma are able to diffuse into the target tissue.

Gumus et al. [37] reported that specific polyphenols such as thymol may reduce plasma lipids by altering the hepatic triglyceride secretion, as well as inhibiting the activity of cholesterol-synthesizing enzyme 3-hydroxy-3-methylglutaryl-coenzyme A reductase. If some imbalance between the cellular free radical formation and the antioxidant defence system exists, excessive amounts of free radicals are produced, and cellular components such as the lipids are attacked. The administration of antioxidants such as thymol could combat oxidative stress by the scavenging of free radicals and, in this way, effectively reduce the serum levels of lipid parameters such as triglycerides and cholesterol [6]. Our study suggests that thymol in the rabbit organism possesses a lipid-reducing function by this mechanism (Table 3).

AST, ALT and ALP are enzymes that significantly reflect the liver function. These parameters in our study were in the range of the reference interval. We assumed that thymol in this concentration was able to express its antioxidant properties and positively affected these parameters, although only ALP was affected significantly. The values of urea and creatinine also remained within the normal ranges. This suggests that no hepatic or renal injuries occurred in this experiment. The slight increase in the urea and creatinine amounts could imply the effect of thymol and/or its metabolites on the kidney function, particularly on glomerular filtration. Oceľová [30] and Kohlert et al. [32] suggested the role of the kidneys in the metabolism and elimination of phenolic compounds. They assumed that thymol metabolites could be cleaved at the brush border of the renal tubule and reabsorbed as thymol back into the peritubular capillaries. As mentioned above, thymol can accumulate in the kidneys, and based on this finding, we can hypothesise that thymol concentrations in the kidneys in our experiment could lead during the metabolic processes to the formation of some toxic substances that could be responsible for altering the renal function (Table 3).

The nutritive value of the protein is determined not only by its amino acid composition but, also, by its digestibility [38]. The antioxidative and antimicrobial properties of thymol may beneficially affect the gastrointestinal microbiota ecosystem and, in this way, improve the nutrient utilisation. Moreover, phytogenics can stimulate the production of digestive enzymes and, also, beneficially affect the nutrient digestibility [39]. Oceľová [30] found that the duodenal wall in chickens was repeatedly exposed to thymol molecules after the four-week addition of thyme essential oil to their diet. Based on the significant correlation between thymol in the plasma and intestinal wall in our experiment, we assumed that the same processes were also present in the rabbit intestinal wall. Many studies have suggested that thymol possesses useful antioxidant properties that are responsible, among other things, for the renewal rate of mature enterocytes at the surface of the intestinal villi, which causes an increase in their absorption capacity [40]. Gasco et al. [41] reported that serum TP synthesis depends on the content of available protein in the diet. We assume that the increased TP in our groups with the thymol addition, as well as thymol withdrawal, was caused by extending the absorption surface of the intestinal wall, which probably evoked a better absorption of the proteins from the diet, and/or also by a higher production of the digestive enzymes (Table 3).

### 4.4. Antioxidant Parameters and LDH in Blood and Tissues

In the present study, the positive effect of thymol on the activity of GPx, which protects intracellular lipids against peroxidation, as well as on MDA levels as an indicator of lipid peroxidation, was expressed in plasma. This finding reflects the fact that thymol was efficiently absorbed from the digestive tract to the blood, where it manifested its antioxidant properties. The antioxidant properties of thymol were also demonstrated in muscles during the thymol addition, as well as after its withdrawal. Oxidative stress is associated with damage in a wide range of macromolecular species and derangements of the cellular metabolism, which permeabilise the cellular or organelle membranes and cause apoptosis [42]. LDH is an intracellular enzyme, and its increased level is an indicator of cellular damage. We detected decreased LDH levels, which points to the sparing effect of thymol on cellular metabolism (Table 4).

### 4.5. Fatty Acids in Muscle

Rabbits need adequate amounts of essential FA, which are primarily represented by linoleic (C18:2, n-6) and α-linolenic acids (C18:3, n-3) in their diet. The stability of these essential FA is very low, and for example, linolenic acid is oxidized ten times more rapidly than linoleic acid [43]. Polyunsaturated FA are easily oxidized because of the unstable double bonds between their carbon atoms and are rapidly broken down into short-chain compounds, which is undesirable from the nutritional aspect. The lipoperoxidation of the cell structure is a consequence of oxidative stress. These biological reactions are initiated by the production of ROS, which remove protons from FA [44]. Dabbou et al. [45] demonstrated that bilberry pomace, which is characterised by high antioxidant activity due to high contents of phenols, effectively prevented the oxidation of unsaturated lipids in the muscles of rabbits. An increase in unsaturated FA and, mainly, linolenic acid were observed in our study after the addition of thymol, which probably blocked the oxidation of the lipids with its strong antioxidative properties. After thymol withdrawal, linoleic acid, as well as ∑n-6, decreased, which could be the reason for the insufficient effect of thymol in connection with its low deposition in the muscles (Table 5). Moreover, as was mentioned above, linoleic acid is more rapidly oxidized than other polyunsaturated FA.

## 5. Conclusions

In conclusion, after its sustained application, thymol is able to accumulate and circulate in the rabbit organism, particularly due to the rabbits′ specific digestive characteristic, i.e., caecotrophy. The investigated parameters were affected not only during the thymol addition to the feed but, also, after its withdrawal, which points to thymol accumulation in the organism. The thymol concentration used in this experiment clearly demonstrated its antioxidant properties and inhibited the lipid peroxidation and formation of oxidative deterioration compounds and may become an important antioxidant food supplement. However, further studies should be conducted to confirm the thymol distribution and deposition within the rabbit organism. Since there is a lack of literature describing the absorption and distribution of thymol in rabbit tissues in connection with its beneficial effects on animal health, to our knowledge, this is the first study attempting to explain these processes in the rabbit organism.

## Figures and Tables

**Table 1:** Ingredients and chemical composition of rabbit diets.

Ingredients (%)		Chemical Composition (g/kg Feed)	
Dehydrated Lucerne meal	36.0	Dry matter (g/kg)	900.9
Dry malting sprouts	15.0	Organic compounds	831.8
Oats	13.0	Nitrogen free extract	444.3
Wheat bran	9.0	Neutral detergent fibre (NDF)	352.9
Barley	8.0	Acid detergent fibre (ADF)	208.1
Extracted sunflower meal	5.5	Crude fibre	177.8
Extracted rapeseed meal	5.5	Crude protein	176.6
Dried distiller grains with solubles	5.0	Cellulose	163.1
Premix ^1^	1.7	Hemicellulose	144.8
Limestone	1.0	Starch	133.1
Sodium chloride	0.3	Ash	69.2
		Fat	33.1
		Metabolic energy, MJ/kg	9.9

^1^ The vitamin-mineral premix provided per kg of complete diet: Retinyl acetate 5.16 mg, Cholecalciferol 0.03 mg, Tocopherol 0.03 mg, Thiamine 0.8 mg, Riboflavin 3.0 mg, Pyridoxin 2.0 mg, Cyanocobalamin 0.02 mg, Niacin 38 mg, Folic acid 0.6 mg, Calcium 1.8 mg, Iron 70 mg, Zinc 66 mg, Copper 15 and Selenium 0.25 mg.

**Table 2:** Thymol content in plasma (µg/L), feed, intestinal wall and faeces (µg/g dry matter (DM)).

Substance	TA	TW
Feed	148.90 ± 16.65	-
Plasma	0.05 ± 0.02	ND
Intestinal wall	0.04 ± 0.03	ND
Faeces	0.89 ± 0.45	0.08 ± 0.04

ND—not detected, TA—thymol addition and TW—thymol withdrawal.

**Table 3:** Effects of thymol on aspartate aminotransferase (AST, µkat/L), alanine aminotransferase (ALT, µkat/L), alkaline phosphatase (ALP, µkat/L), total proteins (TP, g/L), urea (mmol/L), creatinine (µmol/L), triglycerides (mmol/L) and cholesterol (mmol/L) in rabbit blood.

Parameter	TA			*p*-Value	TW			*p*-Value
	C	T	SD		C	T	SD	
AST (µkat/L)	0.45	0.34	0.12	0.1199	0.49	0.34	0.20	0.2948
ALT (µkat/L)	0.77	0.62	0.14	0.2677	0.76	0.69	0.32	1.0000
ALP (µkat/L)	2.00 ^a^	1.52 ^b^	0.42	0.0183	2.42	2.19	0.83	0.5476
TP (g/L)	36.20 ^a^	61.86 ^b^	13.88	0.0025	36.84 ^a^	55.90 ^b^	11.79	0.0079
Urea	5.18	5.31	0.50	1.0000	5.03 ^a^	6.54 ^b^	0.95	0.0079
Creatinine	79.67 ^a^	115.40 ^b^	19.55	0.0357	64.25 ^a^	115.60 ^b^	28.52	0.0159
Triglycerides	1.15	1.45	0.29	0.1508	1.53 ^a^	0.84 ^b^	0.49	0.0317
Cholesterol	2.20 ^a^	1.17 ^b^	0.80	0.0228	1.76	0.95	0.60	0.0556

^a,b^ Values within a row with different superscript letters differed significantly (*p* < 0.05). Data are presented as mean ± standard deviation (SD). C—control diet, TA—thymol addition and TW—thymol withdrawal.

**Table 4:** Effects of thymol on the antioxidant parameters and activity of lactate dehydrogenase (LDH) in rabbit blood, liver and muscle.

Parameter	TA			*p*-Value	TW			*p*-Value
	C	T	SD		C	T	SD	
Blood								
MDA (nmol/mL)	0.35 ^a^	0.254 ^b^	0.06	0.0030	0.32	0.30	0.04	0.1049
GPx (µkat/g Hb)	3.61 ^a^	2.52 ^b^	0.78	0.0177	2.61	2.32	0.71	0.8763
Liver								
MDA (nmol/g protein)	83.11	83.40	18.31	0.7984	83.81	68.69	21.51	0.1605
LDH (µkat/g protein)	41.56	41.62	6.50	0.6454	48.88	42.75	7.83	0.1304
Muscle								
MDA (nmol/g protein)	23.63	28.45	11.20	0.5737	18.34	23.45	5.82	0.0650
LDH (µkat/g protein)	78.74 ^a^	35.89 ^b^	36.47	0.0411	78.50	50.62	43.60	0.2026

^a,b^ Values within a row with different superscript letters differed significantly (*p* < 0.05). Data are presented as mean ± standard deviation (SD). C—control diet, TA—thymol addition, TW—thymol withdrawal, MDA—malondialdehyde and GPx—glutathione peroxidase.

**Table 5:** Effects of thymol on fatty acids profile (% of total FA) in rabbit muscle.

Parameter	TA			*p*-Value	TW			*p*-Value
	C	T	SD		C	T	SD	
C 12:0	0.107	0.109	0.006	0.3428	0.105	0.108	0.004	0.1015
C 14:0	1.383	1.392	0.030	0.5950	1.357	1.386	0.039	0.1375
C 16:0	24.400	24.320	0.159	0.3823	24.390	24.580	0.222	0.1049
C 18:0	10.460	10.550	0.251	0.5049	10.750	10.620	0.210	0.2392
C 17:0	0.302	0.308	0.029	0.3713	0.312	0.286	0.037	0.4001
∑ SFA	36.650	36.680	0.344	1.0000	36.910	36.980	0.280	1.0000
C 18:1 n-7	4.945	4.975	0.109	0.6454	4.854	4.899	0.105	0.3439
C 18:1 n-9	30.030	34.470	3.754	0.0104	31.960	37.270	3.660	0.0006
C 20:1	0.597	0.589	0.091	0.8785	0.545	0.561	0.072	0.5054
∑ MUFA	35.600	40.020	3.730	0.0104	37.420	42.670	4.640	0.2345
C 18:2 n-6	52.59	50.73	6.435	0.5054	51.36	48.83	8.458	0.0070
C 20:4 n-6	1.765	1.978	0.244	0.1049	1.842	1.708	0.279	0.5054
∑ n-6	54.35	52.70	6.341	0.5054	53.20	50.53	4.756	0.0007
C 18:3 n-3	1.794	1.958	0.149	0.0227	1.793	1.885	0.191	0.2786
C 20:5 n-3	1.088	1.108	0.103	0.6350	1.096	0.935	0.173	0.1409
C 22:5 n-3	1.270	1.393	0.127	0.0513	1.276	1.381	0.081	0.0069
C 22:6 n-3	0.328	0.298	0.049	0.3431	0.341	0.310	0.046	0.2677
∑ n-3	4.479	4.755	0.236	0.0519	4.506	4.511	0.199	1.000
∑ n-6/∑ n-3	12.14	11.10	1.444	0.1949	11.86	11.19	1.223	0.3282

Data are presented as mean ± standard deviation (SD). C—control diet, TA—thymol addition, TW—thymol withdrawal, SFA—saturated fatty acids and MUFA—monounsaturated fatty acids.
